# The Pertinence of Microwave Irradiated Coconut Shell Bio-Sorbent for Wastewater Decolourization: Structural Morphology and Adsorption Optimization Using the Response Surface Method (RSM)

**DOI:** 10.3390/ijerph15102200

**Published:** 2018-10-09

**Authors:** Mohammed Abdulsalam, Hasfalina Che Man, Aida Isma Idris, Zurina Zainal Abidin, Khairul Faezah Yunos

**Affiliations:** 1 Department of Biological and Agricultural Engineering, Faculty of Engineering, Universiti Putra Malaysia, UPM Serdang, Selangor 43400, Malaysia; hasfalina@upm.edu.my; 2 Department of Agricultural and Bioresources Engineering, Ahmadu Bello University, Zaria 810222, Nigeria; 3 Department of Chemical Engineering, Segi University, Kota Damansara Selangor 47810, Malaysia; aidaisma@segi.edu.my; 4 Departments of Chemical and Environmental Engineering, Faculty of Engineering, Universiti Putra Malaysia, UPM Serdang, Selangor 43400, Malaysia; zurina@upm.edu.my; 5 Department of Food and Process Engineering, Faculty of Engineering, Universiti Putra Malaysia, UPM Serdang, Selangor 43400, Malaysia; kfaezah@upm.edu.my

**Keywords:** microwave heating, bio-sorbent, characterization, adsorption, colour removal, optimization

## Abstract

Palm oil mill effluent contains carcinogenic coloured compounds that are difficult to separate due to their aromatic structure. Though colour treatment using adsorption processes at lower pH (<4) have been reported effectual, due to its acidity the remediated effluent poses an environmental hazard as a result. Thus, the current study focused on achieving decolourization at neutral pH by enhancing the morphology of the coconut shell activated carbon (CSAC) using N_2_ as activating-agent with microwave irradiation heating. The microwave pretreated and non-pretreated CSAC were characterized using scanned electron microscopy (SEM), energy dispersive X-ray (EDX) and Brunauer-Emmett-Teller (BET) analysis. A significant modification in the porous structure with a 66.62% increase in the specific surface area was achieved after the pretreatment. The adsorption experimental matrix was developed using the central composite design to investigate the colour adsorption performance under varied pH (6–7), dosage (2–6 g) and contact time (10–100 min). At optimum conditions of neutral pH (7), 3.208 g dosage and contact time of 35 min, the percentage of colour removal was 96.29% with negligible differences compared with the predicted value, 95.855%. The adsorption equilibrium capacity of 1430.1 ADMI × mL/g was attained at the initial colour concentration of 2025 ADMI at 27 °C. The experimental data fitted better with the Freundlich isotherm model with R^2^ 0.9851.

## 1. Introduction

The colour compounds (phenolic, carotene, lignin and pectin) in palm oil mill effluent (POME) are carcinogenic and difficult to degrade, due to their benzene and aromatic structure [1]. In addition to this crucial challenge in wastewater management, the continuous discharge of such coloured effluents reduces the solar refraction intensity in a water body, thus reducing photosynthetic activities of the plankton present [2]. This effect could lead to total distortion of the environmental and aquatic ecosystem. In an effort to check these hazardous consequences on the environment, several effluent remediation techniques have been employed such as coagulation [3,4], chemical oxidation [5,6], membrane filtration [7,8], biological decomposition [9,10,11] and adsorption [12]. Among these techniques, adsorption is recognized to be one of most extremely efficient ways of minimizing coloured pigments along with other contaminants such chemical oxygen demand (COD) and total suspended solids (TSS) [13,14,15].

Many agricultural bio-materials have been used as a precursor for preparing biosorbents. The most common ones include palm kernel shell [16,17], coconut shell [18,19], corn stalk [20,21], animal bones [22,23] and shells [24]. The activated carbons produced from the various precursors present distinct adsorption performance, which can be attributed to their activation procedures, the confined carbon density and the structural matrix of the precursors [24]. Specifically, coconut shell is readily available, highly carbonaceous and structurally stable [25]. A report has also shown that coconut shell contains a balanced composition of cellulose, lignin and hemicellulose, which are all carbon-based molecules [26]. Furthermore, such a constitution ensures the efficacy of the carbonation and activation process to generate activated carbon (AC) with a large specific surface area, adsorptive functional groups (such as carboxyl, hydroxyl, carbonyl, etc.), and availability of active sites that favours the adsorption mechanism [27]. To recap, the application of coconut shell activated carbon appears to be one of the cheapest, reliable and effective physicochemical methods for POME treatment [19,26].

Quite a number of works have reported using coconut shell AC to treat POME. Azmi and Yunos [28] investigated the performance of coconut shell AC to alleviate turbidity and TSS from POME. Over 71.26% removal was reported after 35.94 min contact time with a steady agitation speed of 39.82 rpm. Also, Amosa et al. [29] study the performance of high surface area adsorbent to reduce turbidity and TSS, and they reported 89.1 and 91.4% removal efficiency, respectively. In addition, Mohammed and Chong [17] used palm kernel shell activated carbon to decolourize POME under variable contact time, pH and adsorbent dosage. From their report, 100% decolourization was achieved at a pH of 2. Equally, Alkhatib et al. [12] optimize colour removal from POME by granular activated carbon using the response surface method (RSM) and 89.95% decolourization was reported at 4.05 pH. In overview, it can be deduced that the studies using coconut shell biosorbent are rather limited and it has been scarcely applied for POME decolourization despite its impressive potential. More so, the previous reports on the application of CSAC biosorbents for POME decolourization anonymously confirmed that significant colour removal is only attained at an acidic pH [27,30,31,32]. Thus, discharging such partially treated effluent would also also hazardous to the environment because of its acidic pH. It can be deduced from these literatures that the sorbents provide adequate active sites for efficient removal of the colour pigments only if the pH is within the extreme acidic range [17].

In this regards, this study focused on modifying the morphology of the coconut shell biosorbent using microwave irradiation to enhance the specific surface area and pore volume to improve the colour adsorption capacity within the acceptable range of pH (6 to 7). Technically, microwave irradiation induces interior heating as a result of the dipole rotation and ionic conduction of the char particles under the application of a high-frequency voltage [33,34]. More interestingly, this method of heat generation guarantees uniform distribution and minimal loss of energy [35]. Thus, stimulating the expansion of specific surface area, a pore volume as well as increase the adsorption capacity [27]. Afterwards, the pretreated and modified CSAC and the precursor were characterized and compared using scanned electron microscopy (SEM), energy dispersive X-ray (EDX) and Brunauer-Emmett-Teller (BET) analysis. Furthermore, adsorption performance of the microwave pretreated sorbent was studied and optimized using response surface method (RSM) under varied pH, sorbent dosage and contact time conditions. Then, the equilibrium data obtained were fixed into the Freundlich and Langmuir Isotherms models to examine the adsorbent and adsorbate interaction.

## 2. Materials and Methods

### 2.1. POME Samples Preparation

About 50 litres of POME sample was collected from the final discharge outlet of a nearby palm oil milling company at Selangor, Malaysia. The company uses a ponding and facultative treatment method which comprises five ponds with different capacities and distinct retention periods. Degradation of the raw POME takes place sequentially through the ponds to the final discharge outlet, where the POME sample was taken and stored in a VC500 chiller at 4 °C prior to the experimental usage. This procedure was observed to reduce deterioration of chemical and biological properties of the POME sample before the experiment.

### 2.2. Microwave Irradiation Pre-Treatment

Physically activated powdered coconut shell bio-sorbent (CSAC) was procured from Merck KGaA (Petaling Jaya, Selangor Darul Ehsan, Malaysia) and washed vigorously with distilled water to eliminate all impurities. The washing continues till a neutral pH was assumed and then the material was oven dried at 110 °C for a period of 12 h. Then, the microwave reactor was set up to establish a required temperature of 900 °C [36]. Afterwards, about 30 g of the cleaned and dried precursor was placed in a quartz stainless container of the reactor along with 200 cm^3^/min steady flow of the active-agent (N_2_) for a period of 10 min. The microwave pretreated CSAC was allowed to cool to the room temperature inside a desiccator, and then carefully packed into an airtight bottle for further study.

### 2.3. Characterization of the Pretreated and Non-Pretreated AC

The microwave pre-treated and non-pretreated CSAC were characterized using SEM (Model: JIB-4700F; Joel, Peabody MA 01960, USA), EDX (Model: X-123; AMPTEK, DeAngelo Drive Bedford, MA 01730, USA) and BET (Model: 3Flex Version 1.02; Micrometrics, Norcross, GA 30093-2901, USA) analysers. SEM tests were conducted to examine the surface morphology of the adsorbents at 3000× magnification. EDX tests examined the elemental composition of the pretreated and non-pretreated absorbents. The BET surface area and pore volume of the two sorbents were measured based on N_2_ adsorption isotherms using the automated gas sorption system at 77K.

### 2.4. Analytical Procedure

#### 2.4.1. Initial Concentration Analysis

The initial ADMI colour concentration was examined using a DR/4000-U spectrophotometer (HACH, Loveland, CO, USA) by turning on the 1660-HACH programme at 700 nm wavelength to analyse the magnitude. The experiments were conducted in triplicate. All the measurements were conducted following the standard procedure [37].

#### 2.4.2. Adsorption Optimization Experiment

The central composite design (CCD) component of the RSM (free trial of Design Expert 10.0.6.0; Stat-Ease, Godward St NE, Minneapolis, MN, USA) was used to develop the experimental matrix of the batch adsorption study. The adsorption test was conducted at a temperature (27 °C) while the pH (6 to 7), sorbent dosage (2 to 6 g) and contact time (10 to 100 min) where varied accordingly. The adsorption experiments were conducted in a set of plastic Erlenmeyer flasks (250 mL) containing an equal volume of POME sample, 100 mL, following the experimental matrix. The treated samples were placed in the isothermal incubator shaker set at agitation speed and temperature of 150 rpm and 27 °C, respectively. All the treated samples were filtered before the final concentration analysis using 0.47 μm filter paper. After that, the filtrates were used for the final ADMI colour concentration analysis using the DR/4000-U spectrophotometer at 700 nm wavelengths. Each of the experiments was replicated three times to improve the accuracy of the results by taken the average. The summary of the experimental design for the colour adsorption study is shown in Table 1. Subsequently, the percentages of colour removal were determined using Equation (1):

(1) $$\mathrm{Removal}\;\mathrm{Percentage}\;\left(\%\right)=\frac{C_{i}-C_{f}}{C_{i}}\times 100$$

where $C_{i}$ is the initial ADMI colour concentration and $C_{f}$ is the final ADMI colour concentration.

#### 2.4.3. Adsorption Capacity Test

The adsorption equilibrium analysis was conducted using a series of Erlenmeyer flasks of 250 mL capacity containing an equal volume of POME (100 mL) but of variable concentration. The colour concentrations were varied using different dilution factors to attain the initial concentration of 450–2025 ADMI. An equal dose of 10 g of the pretreated CSAC was added to every flask. Then, the samples were subjected to equal pH and agitation speed of 7 and 150 rpm, respectively. The percentage of colour removal in each of the treatment sample was examined at an interval of varied contact time: 10, 20, 30, 40, 50, 60, 70, 80, 90 and 100 min. The adsorption equilibrium capacity ($q_{e}$) of the pretreated bio-sorbent was determined using Equation (2):

(2) $$Adsorption\;equilibrium\;capacity\;\left(q_{e}\right)=\frac{C_{i}-C_{e}}{M}\times v,\;\left(ADMI\times \frac{\mathrm{mL}}{g}\right)$$

where v is the volume of the POME sample, (mL), $C_{e}$ is the equilibrium colour concentration, (ADMI) and M is the mass of sorbent dosage, (g).

#### 2.4.4. Adsorption Isotherms Test

The obtained adsorption experimental data were fitted with the popular Freundlich and Langmuir Isotherms models to examine the interaction of the adsorbent and the adsorbate.

##### The Freundlich Isotherm Model

(3) $$q_{e}=K_{F}C_{e}^{\frac{1}{n}};\;(\log q_{e}\;\mathrm{vs}.\;\log C_{e})$$

where $K_{F}$ is Freudlich constant and it was determined from the intercept, while n indicates heterogeneity of the surface adsorption intensity and it was determined from the slope.

##### The Langmuir Isotherm Model

(4) $$\frac{C_{e}}{q_{e}}=\frac{C_{e}}{q_{0}}+\frac{1}{q_{0}K_{L}};\;(q_{e}\;\mathrm{vs}.\;\frac{q_{e}}{K_{L}C_{e}})$$

where $q_{0}$ is the Langmuir constant determined from the slope of the graph, while $K_{L}$ indicates the adsorption energy and it was determined from the graph intersection.

## 3. Results and Discussion

### 3.1. Effect of Microwave Pre-Treatment on CSAC Morphology

#### 3.1.1. SEM Analysis

**Figure 1:**
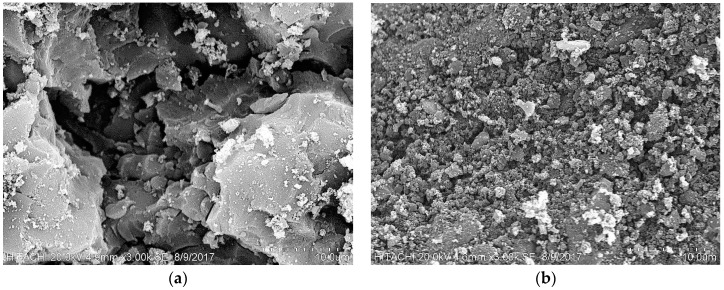
SEM photograph at 3000× magnification of: (**a**) non-pretreated; (**b**) microwave pretreated CSAC.

The distinctive SEM pictures at 3000× magnification of the non-pretreated and microwave pretreated CSAC are presented in Figure 1a,b, respectively. Based on Figure 1a, a flat surface morphology with fewer cracks was observed at the 3000× magnification. The surface cracks and pore formation are very important physical characteristics for a good adsorption process [27]. However, a different scenario was noticed in Figure 1b. From this figure, several cracks were observed on the surface, and the modification in the morphology might be presumed to be due to the microwave pretreatment [36]. According to the previous studies, activated carbon with adequate grainy surface is characterized by a wider ranges of pores sizes, availability of more active sites and larger BET surface area. These features expedite the adsorption mechanism, and also improve the capacity of the adsorbate removal per unit of the sorbent [17,38]. Jalani et al. [38] reported that rapid adsorption mechanism is usually observed at the beginning of the adsorption treatment due to the availability of more bare pores and active sites. This process could lead to the formation of nebulous appearance due to the deposited adsorbate [19,39,40].

#### 3.1.2. EDX Analysis

EDX tests were conducted to compare the intensities of the constituted elements present in the non-pretreated and pretreated CSAC. Figure S1 depicts the intensities of the elements present in the non-pretreated sorbent (see Supplementary Data). The constituent elements include carbon (C), oxygen (O), silicon (Si), chlorine (Cl) and potassium (K) at different percentage compositions, (Table 2). The C was observed to be dominant with 95.03% by weight composition. However, a different pattern EDX elemental intensity and composition were observed after the microwave pretreatment, (Figure S2, Supplementary Data). The carbon (C) composition increased to 98.93%, while the O, Si and K values were reduced considerably. Noticeably, Cl is completely absent in the EDX analysis of the microwave-pretreated CSAC along with the diminished percentage composition of all other constituent elements (O, Si and K, Table 2).

Overall, the increase in C composition was due to interior heating which induced oxidation processes that released volatile matter [41,42]. This resulted in the gross differences in the weight composition and elements contained in the pretreated and non-pretreated CSAC [41]. Furthermore, the induced internal heating also accounted for the surface metamorphosis and the formation of more porous structure, cleavages as well as the aperture on the pretreated CSAC to give a better platform for adsorption process [43].

#### 3.1.3. BET Analysis

**Figure 2:**
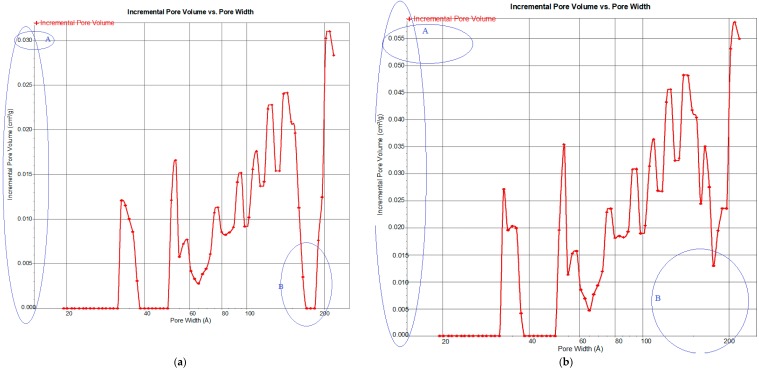
Incremental pore volume versus pore width of the (**a**) non-pretreated and, (**b**) microwave pretreated CSAC.

A Micrometric 3Flex Version 1.02 model instrument was used to examine the pore size distributions and the sorbent-specific surface areas (S_BET_) based on isotherm of N_2_ adsorption at 77 K. Figure 2a,b present the incremental distributions of pore volume (P_v_) with respect to the pore width of the non-pretreated and the microwaved pre-treated CSAC, respectively. As pointed out in the figures, a significant increase in pore volume was noticed after the microwave irradiation pretreatment. The indicating mark ‘A’ on the vertical axis of the figures signifies a comparative P_v_ of both non-pretreated and pretreated CSAC, and the magnitude was 0.030 cm^3^/g and 0.056 cm^3^/g, respectively. Also, the index ‘B’ on the horizontal axis emphasised that more pores have been created after the microwave irradiation treatment, and this explained the increased P_v_. Therefore, the additional pores generated and widening of the initial pores accounted for the significant increase in the P_v_, (Table 3).

From the figures, it can be deduced that mesoporous (20–100 Å) and macroporous (>100 Å) size distributions are common in the two sorbents (non-pretreated and pretreated). However, a considerable increase in the mesopore and macropore volume was observed after microwave irradiation heating (Figure 2b). In a more wider scope, releasing of the volatile matters and structural rearrangement during the microwave irradiation heating justify the noticed P_v_ increment as well as the formation of new pores [36]. This concurs with the findings reported by Yang et al. [36]. They confirmed that microwave heating contributes to the progressive creation of ultramicropores and this accounted for the overall increase in the P_v_. More so, other studies reported by Yang et al. [44], Foo et al. [45] and Foo et al. [46], are all in agreement with this remark.

Additionally, a considerable increase in the specific surface area (S_BET_) was also observed (Table 3). The S_BET_ of the non-pretreated CSACs increased from 421.5787 to 702.4341 m^2^/g after the pretreatment. Collectively, the increase in the P_v_ and the S_BET_ confirm the efficacy of the microwave irradiation pretreatment. The pretreatment promotes a synchronization effect to accelerate the particles’ agitation, and this results in the generation of internal heat. More interestingly, this method of heat generation guarantees a uniform distribution and minimal loss of energy [35]. Thus, stimulating the expansion of S_BET_, P_v_ as well as increase the adsorption capacity [27].

Ultimately, the microwave irradiation heating rearranges the structure of the carbon to form a more orderly and porous matrix, thereby widening the pores and increasing the surface area [36]. In recap, the pore enlargement and regeneration take place in four distinct phases: widening of the remote pores, restructuring to create new pores, expansion of existing pores and unification of the current pores due to collapsing of the pore wall [24,32]. The induced internal heat engaged the cellulose, lignin and hemicellulose content in the precursor to undergo dehydration, bond breakage and reformation as well as polymerization to create a more porous and larger surface area [47]. Thus, these contribute significantly to the rapid size reduction of the precursor and formation of evenly distributed pores, (Figure 2b). This infers that the microwave irradiation heating forms a useful basis for further improving the porous structure of the activated carbon through the internal heating in presence of the activating agents, such as N_2_ [48].

A comparison appraisal of the surface morphology of the sorbents (non-pretreated and pretreated CSAC) of the current study with other literature values previously reported under optimum activation conditions are presented in Table 4. It is obvious that the S_BET_ of the microwave pretreated CSAC is much higher. The previously reported coconut shell sorbents were only activated using the physical method of activation while the present studies enhanced the morphology of the procured sorbent using microwave irradiation heating. It can be deduced that the enhancement of the specific surface area and the significant change in the morphology were due to the microwave pretreatment through a progressive widening of the ultra micropores and regeneration of more pores [36]. The temperature limit (900 °C) applied for the microwave pretreatment in this study was adopted from Yang et al. [36], and they reported a very high S_BET_ with a value of 2194 m^2^/g (Table 4). According to the authors, the resulting large S_BET_ was due to the double stage activation processes along with the type of agents used [36]. The double stage activation technique requires a temperature of 1000 °C to proceed the carbonation process under a steady supply of N_2_ (active agent) [36]. The second stage involves microwave heating of the carbonated precursor at 900 °C. However, these processes amounted to excessive application of energy and modus complication, even though higher S_BET_ was obtained.

### 3.2. Adsorption Experiments using Microwave-Pretreated CSAC

#### 3.2.1. Effect of pH

**Figure 3:**
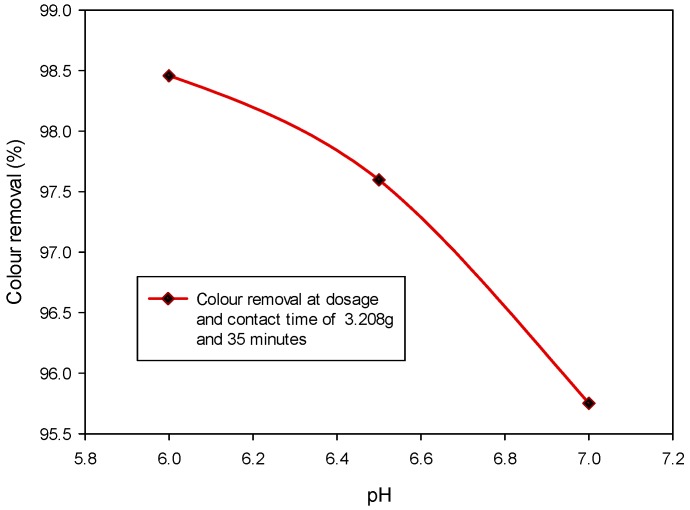
Colour removal from POME under variable pH at fixed sorbent dosage and contact time.

It is an established fact that lower pH has a strong positive effect on colour adsorption [12]. Nonetheless, the impact of the discharged treated acidic wastewater could directly hamper the aquatic habitat. In view of this, the adsorption studies using the pretreated CSAC were conducted within the acceptable pH range of 6 to 7. The other two factors considered are dosage and contact time, and they were maintained at 3.208 g and 35 min, respectively. From Figure 3, it can be observed that the percentage of colour removal increases with a decrease in pH. The highest colour removal of 98.46% was obtained at pH 6 but decreased to 95.69% as the pH increases to 7. Basically, the colour pigments in POME such as the phenolic, pectin and lignin, dissociate to generate negatively charged radicals in the acidic solution [59]. On the other hand, the acidic media favours the oxidation of the pretreated CSAC to acquire positive charges on the surface. This resulted in electrostatic attraction between the positively charged carbon sites (pretreated CSAC) and the ions of the adsorbate [45]. This inference is in agreement with the previous studies [42,60]. Mohammed [61] varied the pH between 2 to 12, and the author observed that the percentage of colour removal increases with a decrease in pH. Similarly, Gupta et al. [62] and Sia et al. [63] reported a comparable study, they also substantiated that lower pH promotes efficient adsorption process. More so, the pH effect is also valid in the adsorption of chromium from the dye, and that pH less than 5 favours efficient removal of the heavy metal [62]. Furthermore, Yang et al. [64] investigated the effect of pH (2 to 9) on Ni(II) reduction using carbon-based nanotube and they concluded that the adsorption capacity decreases with increase in pH. In overall, it can be deduced that as the pH increases, the positivity of the CSAC reduces, thus the electrostatic attraction between the sorbent and adsorbate diminishes.

#### 3.2.2. Effect of Adsorbent Dosage

**Figure 4:**
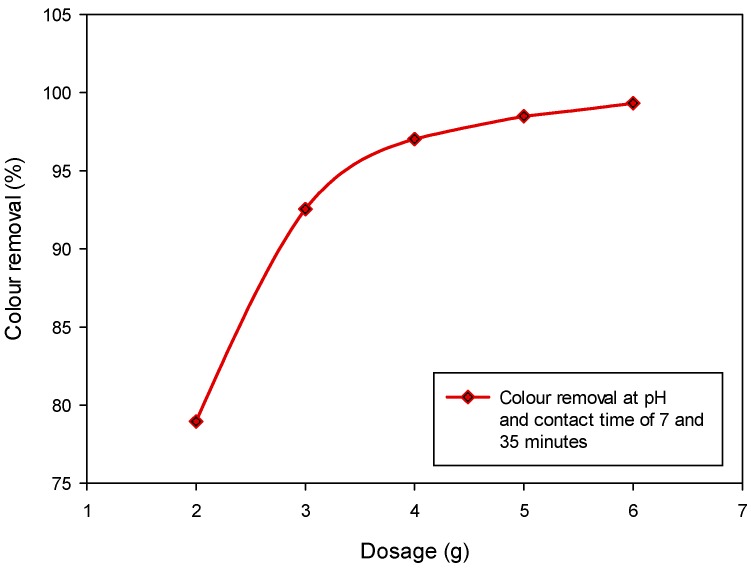
Colour removal from POME under variable sorbent dosage at fixed pH and contact time.

The pretreated CSAC dosage was varied between 2 to 6 g and an equal volume of 100 mL of the POME samples was used throughout the experiments. The pH and contact time were fixed at 7 and 35 min, respectively. It was observed that the percentage of colour removal increases as the dosage increases (Figure 4). The highest colour removal percentage of 99.32% was obtained at 6 g CSAC dosage. The higher performance observed with the greater dosages could be attributed to the availability of more active sites for complete adsorption of the colour pigments [5,17]. This shows that greater dosage provides higher adsorption capacity for efficient colour removal. Noticeably, at the dosage of 4.7 to 6 g, the percentage of colour removal remains relatively constant (Figure 4). This denotes that the adsorption equilibrium has been reached within this range of the dosage. Hence, a further increase in dosage is insignificant for the colour removal.

#### 3.2.3. Effect of Contact Time

**Figure 5:**
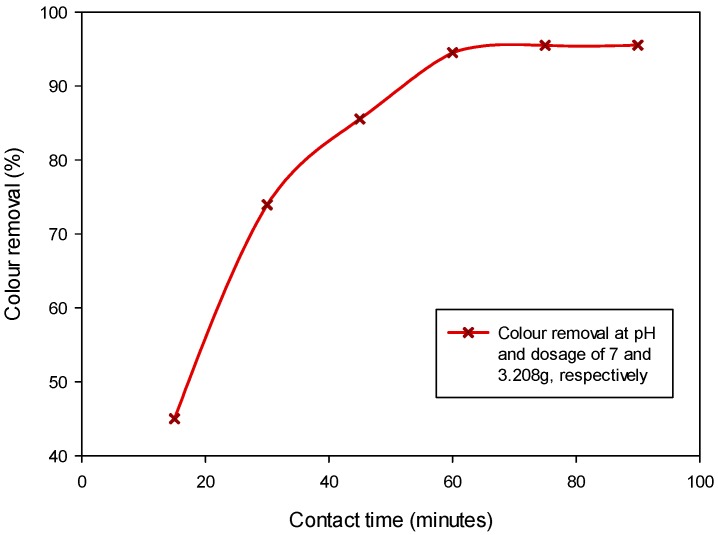
Colour removal from POME under variable contact time at fixed pH and sorbent dosage.

The influence of contact time on the colour removal was observed between 10 to 100 min under the fixed condition of 7 and 3.208 g for pH and CSAC dosage, respectively. It was observed that the rate of colour removal is influenced by the contact time, but became relatively steady after 58 min (Figure 5). 

During the contact time between 10 to 30 min, the process of colour adsorption was rapid but became slow at the contact time was extendedf. The faster adsorption at the beginning of the treatment might be due to the prominence of active sites but became sluggish as the sites got filled with the adsorbate [5,19]. The recorded low colour removal percentage at shorter contact times could be due to the inadequacy of the interaction period for complete adsorption, while, at longer contact times, the available active sites have sufficient time to entrap all the adsorbate, thus better colour removal percentage is attained. On the whole, the active pores gradually got filled with adsorbate (colour pigments ions) until the point of saturation, where the adsorption and desorption rate is equal [36]. Therefore, further extension of the contact time has an insignificant effect on the colour removal [17].

### 3.3. Adsorption Optimization and Validation Result

#### 3.3.1. Result Analysis

The detailed analysis of variance (ANOVA) of the results for colour removal by adsorption under variable treatment conditions of pH (A), dosage (B) and contact time (C) is presented in Table 5. The analysis has a modal F-value of 13.97, which infer a significant model. In accordance with the analysis: the factors B, B^2^ and AC were significant in the regression model for the colour removal with F-value of 89.06, 27.73 and 8.28, respectively (Table 5). Based on the magnitude of the F-value, B has the most significant effect on the colour adsorption, followed by the quadratic term B^2^.

The regression model for the colour adsorption mechanism was developed based on these factors (*B*, *B^2^* and *AC*) with a correlation coefficient (R^2^) of 0.9263, (Table 5). Essentially, this implies that the identified factors (*B*, *B^2^* and *AC*) have a 92.63% influence on the colour adsorption process. More so, the regression model confirmed that the R^2^ is strongly in agreement with the adjusted-R^2^ of 0.8601 for the colour adsorption. Thus, as the R^2^ tends to unity, the model accuracy improves. Hence, the model estimated (colour removal) and experimental values will be closely predicted with insignificant deviations. The regression model in requisites of the significant factors is presented in Equation (10).

(10) $$Colour\;removal=97.38+7.47\times B-2.97\times AC-4.06\times B^{2}$$

**Figure 6:**
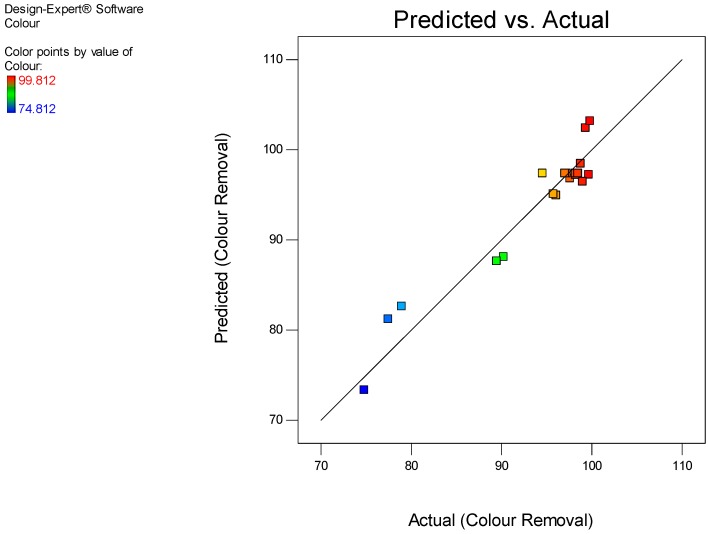
Experimental actual vs. predicted values of colour removal.

It is worth mentioning that the coefficients associated with the factors in the regression model (Equation (10)) denote the effect, while the ascribed positive or negative signs shows its synergistic or antagonistic influences on the colour adsorption, respectively [12,27]. The predicted colour removal was determined using the model equation, and then the obtained values were plotted against the actual values using the RSM diagnostic tool, (Figure 6). From this figure, both the actual and predicted values have a good correlation and the error of deviation between the diagonal line and data points (actual and predicted values) all expressed strong agreement. Therefore, the regression models can be used to navigate the designed space.

#### 3.3.2. Result Validation

Furthermore, the recommended optimal treatment conditions by the software (Design Expert 10.0.0) based on the set criteria were used to validate the regression model obtained. Basically, the optimization criteria applied in this study was to maximize colour removal and the pH (A) value to neutral range, while the sorbent dosage (B), and contact time (C) were minimised. Based on these criteria, the first five (5) experimental solutions suggested by the model were selected and verified in the lab to substantiate the model prediction, the results were presented in Table 6. The average values of the predicted and experimental colour removal are also shown in the table. The average experimental actual value for the colour removal efficiency was 96.292, and it is in close conformity with the model predicted values of 95.855 with the insignificant disparity of 0.4372.

**Figure 7:**
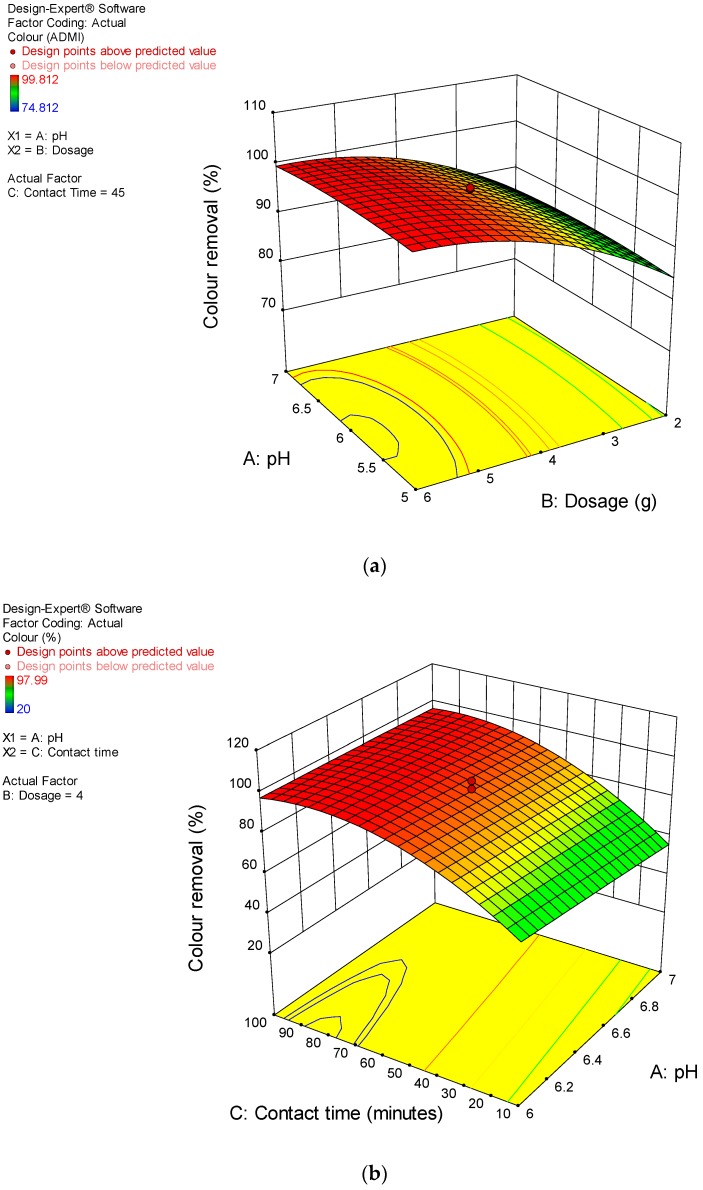
Colour removal from POME under variable: (**a**) dosage, B and pH, A; (**b**) contact time, C and pH, A.

Furthermore, Figure 7a presents the surface response of the effect of CSAC dosage (B) on colour removal. It is obvious that at a lower dosage, the colour removal was low. On the contrary, higher colour removal was observed at the greater dosage, this might be due to the extra availability of active sites for complete adsorption, as reported earlier. Figure 7b also shows the combined effect of A and C, it was noticed that the two factors exert a mutual reciprocal influence on the colour removal. That is, longer contact time (C) and lower pH (A) favours efficient colour adsorption. The observations concur with the previous studies reported [5,12,17,19].

### 3.4. Adsorption Equilibrium Capacity

**Figure 8:**
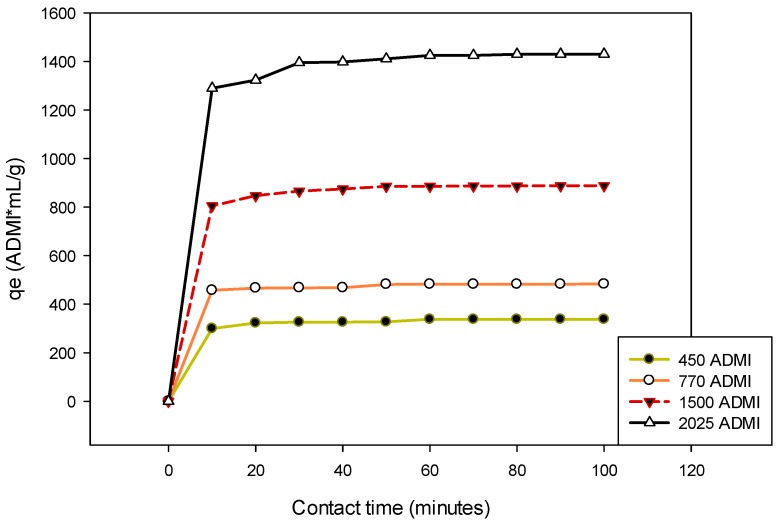
Change in adsorption capacity with contact time at a varied initial concentration (at 27 °C).

The adsorption capacity of the modified CSAC for colour removal from POME was investigated at a variable initial concentration of 450 to 2025 ADMI. The concentrations were varied using dilution factors by adding the appropriate amount of distilled water. Essentially, the experiments were conducted at 27 °C for a period of 100 min to ascertain the equilibrium adsorption for each of the treatment. The obtained results of the equilibrium experiments are presented in Figure 8. It was observed that a contact time between 10 to 30 min is adequate for the 450–770 ADMI initial concentration to attain equilibrium adsorption while a longer time is required for a higher concentration. The samples with an initial colour concentration of 1500–2025 ADMI reached equilibrium adsorptions after 40–60 min of contact time. Conspicuously, the adsorption process proceeds very rapid at the beginning but became slower after a certain period of time (30 min), (Figure 8). Immediately after 40 min of contact time, the rates of colour removal for all the observed concentrations were relatively steady. At this point, a dynamic equilibrium has established and no further significant removal of colour [65]. Thus, the amount of colour removal at the dynamic equilibrium indicates the adsorption capacity of the bio-sorbent. The amount of the adsorption capacity (*q_e_*) increased from to 337.5 to 1430.1 ADMI × mL/g as the initial concentration varied for 450 to 2025 ADMI. This shows that the magnitude of the removal percentage depends on the initial colour concentration of the POME sample. This might be due to the rapid collision between the adsorbate and the sorbents as the concentration increases [66].

### 3.5. Adsorption Isotherm

The experimental data of the adsorption equilibrium capacity were validated using the popular Freundlich and Langmuir isotherm models based on a linear regression technique. Basically, the fitting of the experimental data into the isotherm models assisted in describing the adsorption process using the model’s constants and coefficients of correlations (R^2^). This was achieved by plotting log *q_e_* against log *C_e_* based on the linear form of the Freundlich model, while *C_e_*/*q_e_* was plotted against *C_e_* in accordance with the linear expression of the Langmuir model. The constants (*K_F_*, 1/*n*, *q*_o_, and *K_L_*) were determined from the gradients and interceptions of the isotherms plots (Figures S3 and S4, supplementary data) along with the fitness correlation R^2^, the summaries of the results are presented in Table 7.

The Freundlich isotherm model assumes heterogeneous surface absorption and it describes the sorption from a liquid media onto the solid interface [67]. The important Freundlich constants normally used to describe the nature of the sorption process include *n* and *K_F_*, and they were determined from the slope and intercept of Figure S3 (Supplementary Data), respectively. Essentially, the constant n denotes the heterogeneity of the sorption process such that a magnitude of less than 1 (*n* < 1), equal to 1 (*n* = 1) or greater than 1 (*n* > 1) indicates the prevalence of chemical, linear or physical adsorption, respectively [36]. In addition, the reciprocal of the constant (1/*n*) implies normal sorption; meaning that the adsorption process is favourable [68]. In the current study, the values of *n*, 1/*n*, *K_F_* and R^2^ were 1.031, 0.9758 and 0.9851, (Table 7). Based on this result, it can be deduced that the prevalence of normal adsorption is favourable and the high R^2^ value (0.9851) suggested excellent fit with the Freundlich isotherm model, (Figure S3, Supplementary Data).

Langmuir isotherm model assumes a definite amount of active sites for the adsorption process and that the sorption is only in a monolayer with constant adsorption energy [68]. The K_L_ and q_o_ are the essential constants and they were determined from the intercept and slope of Figure S4 (Supplementary Data), respectively. The *K_L_* is related to the efficacy of the active sites to bind the adsorbate. Thus, higher *K_L_* implies stronger adhesion of the adsorbate on the surface of the adsorbent [12]. In the current study, the value of the *K_L_* was 0.7257, which seems low and may not favour strong binding of the adsorbate onto the activated carbon. More so, the *q*_o_ and the model correlation (R^2^) with experimental data were 0.00003 g/mL and 0.0127, respectively. The low values further confirmed that the Langmuir model was less favourable for the adsorption of the colour from POME solution at 27 °C, as clearly shown in Figure S4 (Supplementary Data).

## 4. Conclusions

It appears that microwave irradiation heating is a prospective technique for enhancing the morphology of a biosorbent. The physically activated coconut shell sorbent was pretreated using microwave irradiation heating and applied for colour removal from palm oil mill effluent (POME). SEM analysis of the resulting sorbents revealed that more cracks and porous structures were created after the pretreatment. The transformation was due to the microwave heating which prompted the precursor to undergo structural changes. This justifies the crack formation along with the increase in the carbon composition from 95.03 to 98.93%, (EDX analysis). The microwave irradiation pretreatment shows excellent efficacy in the increase of the S_BET_ as well as the P_v_. Over 66% increase in SBET was achieved in addition to the 86.67% P_v_ increment. Furthermore, the performance of the pretreated sorbent for colour removal from POME was examined using RSM. The regression model predicted the colour removal with good accuracy of 0.9263 R^2^. Noticeably, as the pH decreased, the percentage of colour removal increased. Similarly, a higher sorbent dosage provides more active sites and this favours efficient colour removal. Undert the optimum treatment condition of 3.208 g dosage, pH of 7 and 35 min of contact time, the average colour removal was 96.29%. The adsorption equilibrium of 14301.1 ADMI × mL/g was obtained at the highest initial concentration of 2025 ADMI. The obtained equilibrium data were fitted with Freundlich and Langmuir isotherm model, it was observed that the Freundlich Model fitted better with the data with a 0.9851 correlation (R^2^). Thus, this study verified that microwave irradiation heating can enhance the morphology of coconut shell sorbent, and the adsorption process followed a Freudlich isotherm model at 27 °C.

## Figures and Tables

**Table 1:** Summary of the experimental design for colour adsorption by the pretreated CSAC.

Independent Factor	Unit	Symbol	Coded Level		
			−1	0	+1
pH	-	A	6	6.5	7
Dosage	g	B	2	4	6
Contact time	minutes	C	10	55	100

**Table 2:** Elemental composition of microwave pretreated and non-pretreated CSAC as determined by the EDX.

Element	Non-Pretreated CSAC		Microwave Pretreated CS-AC	
	Weight %	Atom %	Weight %	Atom %
C	95.03	97.58	98.93	99.36
O	1.56 S	1.20	0.41 S	0.28
Si	0.69	0.30	0.27	0.15
Cl	1.78	0.62	-	-
K	0.94	0.30	0.39	0.21
Total	100.00	100.00	100.00	100.00

**Table 3:** Summary of the BET analysis for both microwave pretreated and non-pretreated CS-AC.

Parameters	Abbreviation	Non-Pretreated CSAC		Microwave Pre-Treated CSAC	Percentage of Increment %
BET Surface Are (m^2^/g)	S_BET_	421.5786		702.4341	66.62
Pore volume (cm^3^/g)	P_V_	0.030		0.056	86.67
**BET Analysis Conditions**					
Analysis adsorptive			N_2_		
Temperature (K)			77		
Cold free space (cm^3^)			60.8085		
Warm free space (cm^3^)			16.2270		
Equilibration interval (s)			10		
Ramp rate (°C/min)			10		
sample density (g/cm^3^)			1.000		

**Table 4:** Comparison of coconut shell sorbent specific surface area of the current study and literature data at optimum conditions.

Reference	Activation Method	Activation Temperature (°C)	Activation Agent	Activation Time (Minutes)	S_BET_ (m^2^/g)
Current Study	Physical	850	Steam	120	421.5786
	Physical + Microwave	900	Steam + N_2_	10 (microwave)	702.4341
Yang et al. [36]	Microwave	900	Steam + CO_2_	75	2194
Su et al. [49]	Physical	850	-	60	663
Monsalvo et al. [50]	Physical	800	CO_2_	240	97
Li et al. [51]	Physical	850	N_2_ + Stream	60	280
Namasivayam and Kadirvelu [52]	Physical	400	Steam	60	346
Hidayu et al. [53]	Physical	765	Steam	77	720
Li et al. [54]	Physical	850	N_2_ + Steam	60	130
Hesas et al. [55]	Physical	500	N_2_	120	484
Singh et al. [56]	Physical	200-800	Inert-Atmosphere	60	378
Achaw and Afrane [57]	Physical	800	Steam + N_2_	120	524
Velghe et al. [58]	Physical	450	N_2_	90	60

**Table 5:** Analysis of variance for the response surface of the quadratic model.

Source	Sum of Squares	df	Mean Square	F Value	*p*-Value Prob > F	
Model	1075.08	9	119.45	13.97	0.0001	significant
*A-pH*	*0.025*	*1*	*0.025*	*2.926E-003*	*0.9579*	
*B-Dosage*	*761.31*	*1*	*761.31*	*89.06*	*<0.0001*	
*C-Contact Time*	*0.18*	*1*	*0.18*	*0.021*	*0.8870*	
*AB*	*1.46*	*1*	*1.46*	*0.17*	*0.6878*	
*AC*	*70.79*	*1*	*70.79*	*8.28*	*0.0165*	
*BC*	*0.11*	*1*	*0.11*	*0.013*	*0.9118*	
*A^2^*	*9.99*	*1*	*9.99*	*1.17*	*0.3050*	
*B^2^*	*237.07*	*1*	*237.07*	*27.73*	*0.0004*	
*C^2^*	*0.24*	*1*	*0.24*	*0.028*	*0.8710*	
*R^2^*	*0.9263*					
*Adjusted-R^2^*	*0.8601*					

**Table 6:** Results of the regression model validation by experiments.

Recommended Optimal Treatment Conditions					Actual Colour Removal	Deviations
Number	pH	Dosage	Contact Time	Predicted Colour Removal		
1	7.000	3.208	35.000	95.844	96.52	0.676
2	7.000	3.193	35.000	95.767	96.21	0.443
3	7.000	3.190	35.000	95.753	96.03	0.277
4	7.000	3.170	35.000	95.649	95.81	0.161
5	7.000	3.289	35.000	96.261	96.89	0.629
Average Values				95.855	96.292	0.437

**Table 7:** Important constants of the isotherm models and fitness correlation.

Isotherm Model	Plotted Variables	Constants			R^2^
		1/*n*	*n*	*K_F_*	
Freundlich: $\log q_{e}=\log K_{F}+\frac{1}{n}\log C_{e}$	$\log q_{e}\;\mathrm{vs}.\;\log C_{e}$	0.9758	1.031	0.8744	0.9851
Langmuir: $\frac{C_{e}}{q_{e}}=\frac{1}{K_{L}q_{o}}+\frac{1}{q_{o}}C_{e}$	$\frac{C_{e}}{q_{e}}\;\mathrm{vs}.\;C_{e}$	1qo	1KL	$K_{L}$	0.0127
		0.00003	1.3780	0.7257	

## Supplementary Materials

The following are available online at http://www.mdpi.com/1660-4601/15/10/2200/s1, Figure S1: EDX analysis showing the elemental composition in the non-pretreated CSAC, Figure S2: EDX analysis showing the elemental composition in the microwave pretreated CSAC, Figure S3: Freundlich isotherm model of colour adsorption onto the pretreated CSAC at 27 °C, Figure S4: Langmuir isotherm model of colour adsorption onto the pretreated CSAC at 27 °C.
