# Supplementary material for: The Pertinence of Microwave Irradiated Coconut Shell Bio-Sorbent for Wastewater Decolourization: Structural Morphology and Adsorption Optimization Using the Response Surface Method (RSM)
Source: Int J Environ Res Public Health. 2018 Oct 9;15(10):2200. doi: 10.3390/ijerph15102200 (PMC6209996; doi:10.3390/ijerph15102200)
Supplement: Supplementary file 1 [file ijerph-15-02200-s001.pdf]

The Pertinence of Microwave Irradiated Coconut Shell Bio-Sorbent for Wastewater Decolourization: Structural Morphology and Adsorption Optimization Using Response Surface Method (RSM)

Supplementary Data

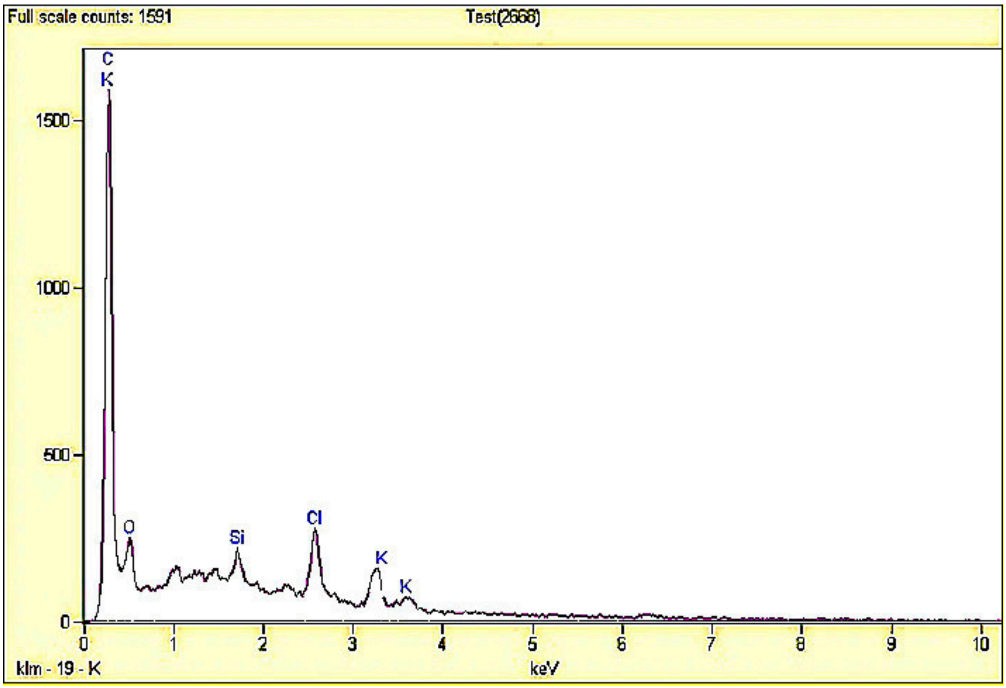

Figure S1. EDX analysis showing the elemental composition in the non-pretreated CSAC.

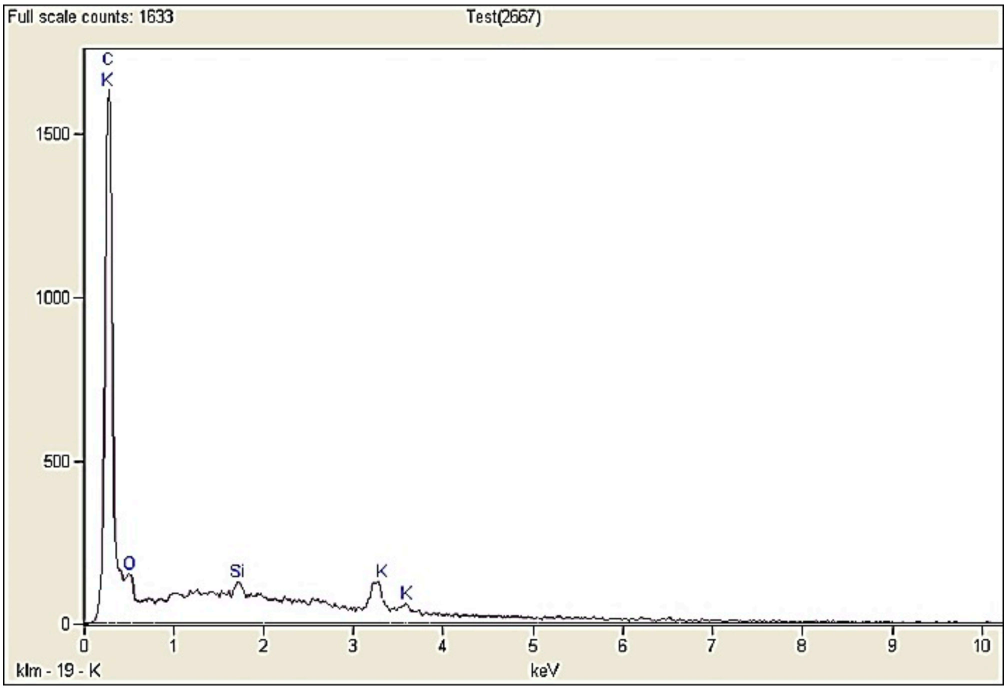

Figure S2. EDX analysis showing the elemental composition in the microwave pretreated CSAC.

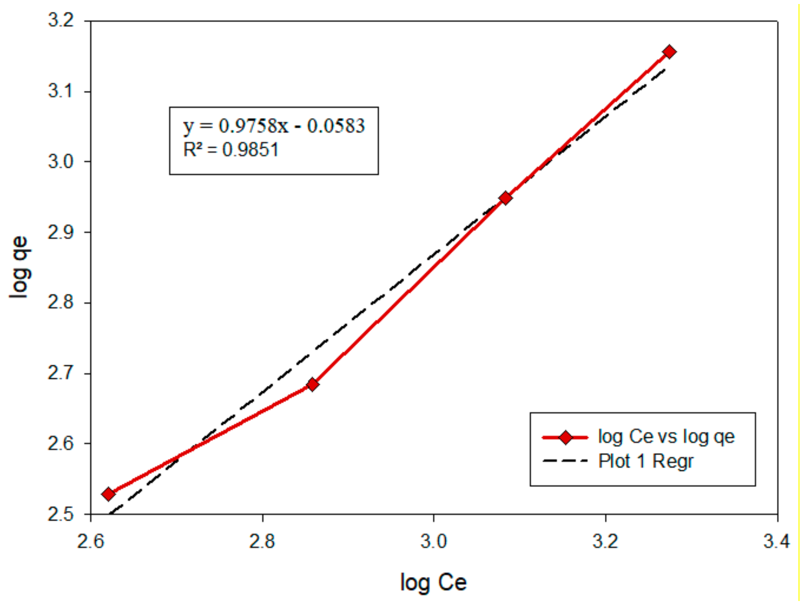

Figure S3. Freundlich isotherm model of colour adsorption onto the pretreated CSAC at 27 °C.

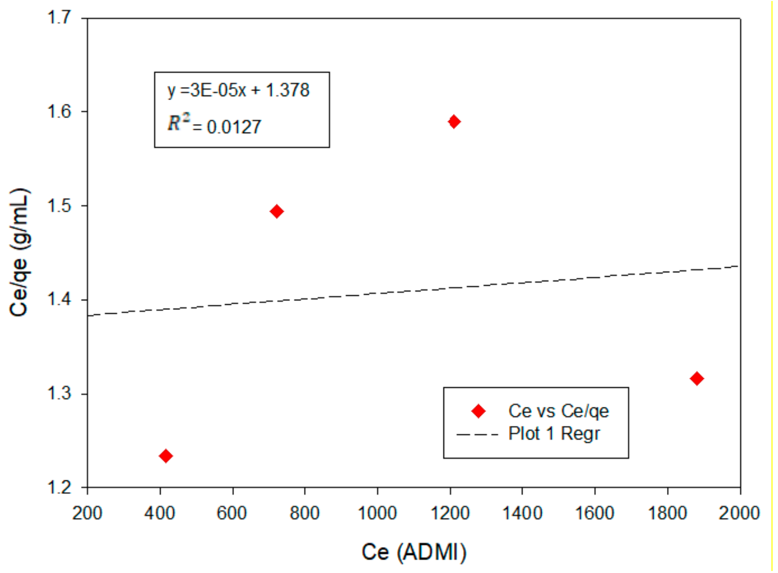

Figure S4. Langmuir isotherm model of colour adsorption onto the pretreated CSAC at 27 °C.
